# Successful therapy of chimeric antigen receptor T cells for isolated extramedullary acute lymphoblastic leukemia

**DOI:** 10.1002/jha2.411

**Published:** 2022-03-24

**Authors:** Xiangqun Li, Kylan Chen, Xian Zhang, Junfang Yang, Jianwei Zheng, Fei Dong, Yongbo Zhu, Jiao Yu, Peihua Lu, Bo Chen

**Affiliations:** ^1^ Kecellitics Biotech Company Ltd Beijing China; ^2^ College of Natural Sciences University of Texas Austin Texas USA; ^3^ Lu Daopei Hospital Langfang Hebei China; ^4^ Lu Daopei Institute of Hematology Beijing China

1

Despite many clinical trials of CD19‐targeted CAR T cells for the treatment of B‐cell acute lymphoblastic leukemia (B‐ALL) having been reported, few studies of CD19 CAR T therapy for isolated refractory/relapsed (r/r) extramedullary ALL (EM‐ALL) patients have been published. Extramedullary involvement is considered an unfavorable prognostic factor, which often reduces leukemia response to induction chemotherapy and is usually associated with shorter progression‐free survival (PFS) and overall survival (OS) [1, 2]. There is still no standard guideline on the treatment of isolated EM‐ALL [3]. Considering the absence of leukemia blasts in the peripheral blood and bone marrow in isolated EM‐ALL patients, the questions of whether CAR T cells can successfully expand in these patients and whether these patients can benefit from CD19 CAR T therapy remain unanswered. In this study, we evaluated the safety and efficacy of CD19 CAR T cells for isolated r/r EM‐ALL patients (ChiCTR2000038532).

Our cohort enrolled nine refractory or relapsed isolated EM‐ALL patients admitted to Hebei Yanda Lu Daopei Hospital between 2019 and 2021. The median age was 14 years old (ranging from 8 to 33). Among them, three isolated EM‐ALL patients were confined to just CNS involvement. The other patients had more complicated EM involvement including liver, bone, lung, neck, or testis. The detailed characteristics are summarized in Table 1. Following leukapheresis, all patients received fludarabine (30 mg/m^2^ per day) and cyclophosphamide (250 mg/m^2^ per day) lympho‐depleting chemotherapy for three consecutive days before the CD19 CAR T cell infusion. As for the patients with CNS infiltration, intrathecal chemotherapy was performed to reduce blasts in the cerebrospinal fluid (CSF) before CAR‐T cell infusion. On day 0, all patients received CAR T cell infusion (3 × 10^5^ to 10 × 10^5^ per kg body weight). The doses were determined based on our previous studies and are described in Table 1. Once complete remission (CR) was achieved, some patients received consolidative hematopoietic stem cell transplantation (allo‐HSCT).

**TABLE 1:** Patient characteristics and clinical outcome of CAR T infusion

Patient	Age (Years)	Gender (F/M)	Site of extramedullary involvement	Previous transplant (Y/N)	WBC at diagnosis (10^9^/L))	Fusion gene/mutation	Refractory/relapsed	Cell dose (×10^5^/kg)	Response after CAR T cell infusion (days)	EMDs elimination (Y/N)	Best Response/LFS (months)
1	12	F	bone, diffused	N	9.61	None	Relapsed	3	CR /MRD‐ (28)	Y	CR/7
2	33	F	liver, CNS, diffused	N	6.36	None	Relapsed	3	CR /MRD‐ (28)	Y	CR/5
3	8	F	CNS	N	2.63	None	Relapsed	3	CR /MRD‐ (28)	Y	CR/12
4	12	M	CNS	Y	6.32	TEL‐AML1	Relapsed	10	CR /MRD‐ (28)	Y	CR/24
5	13	M	CNS, testis	N	3.83	None	Relapsed	3	CR /MRD‐ (28)	Y	CR/24
6	14	M	CNS	Y	5.92	None	Relapsed	10	CR /MRD‐ (28)	Y	CR/27
7	14	F	bone, diffused	N	6.41	None	Relapsed	10	CR /MRD‐ (28)	Y	CR/15
8	28	M	testis, diffused	N	5.36	TP53	Relapsed	10	CR /MRD‐ (28)	Y	CR/4
9	31	M	CNS, liver	N	3.84	None	Refractory	3	CR /MRD‐ (28)	Y	CR/17

Abbreviations: CAR T, chimeric antigen receptor; CR, complete remission; EMDs, extramedullary disease; F, female; LFS, leukemia‐free survival; M, male; MRD, minimal residual disease; N, no; ND, not detected; WBC, white blood cell count; Y, yes.

Robust expansion and persistence of genetically modified T cells in vivo are critical for durable clinical remissions in patients with hematologic malignancies [4]. Previous studies report that exposure to CD19 protein in vivo can trigger the proliferation of CD19 CAR T cells [5]. Therefore, we first evaluated the expansion and persistence of infused CAR‐T cells in the blood by flow cytometry (FCM). As shown in Figure 1A, infused CAR‐T cell expansion peaked from day 7 to 17 in peripheral blood (PB). The median peak of the CAR T cells as a percentage of total lymphocytes was in the range of 3%–33.29%, and a rapid decline followed. The results indicated that CD19 CAR T cells had successfully expanded in most of the isolated EM‐ALL patients, although at a comparatively lower level than those with leukemia blasts in bone marrow (BM) or PB or both (data not shown).

**FIGURE 1:**
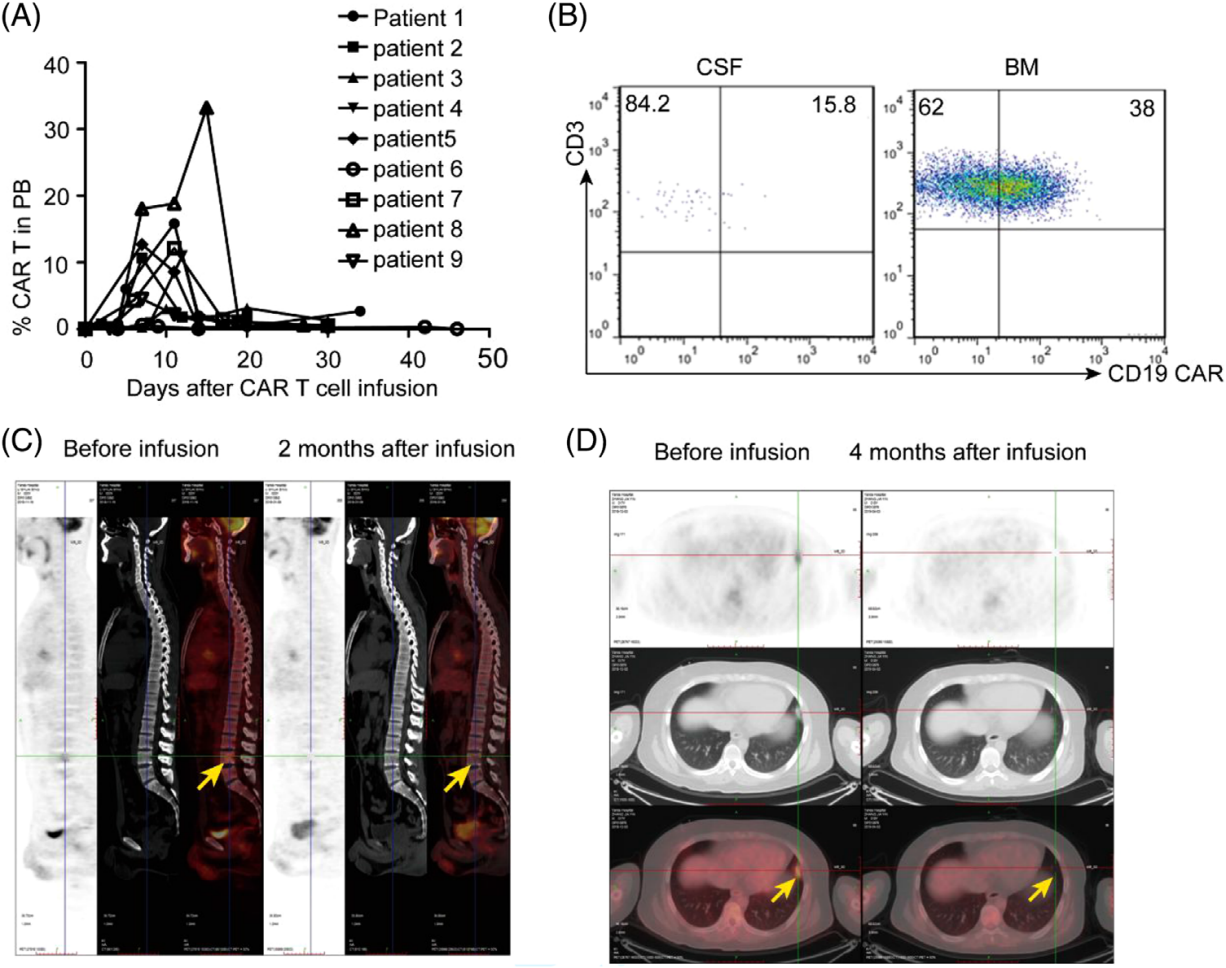
Expansion, persistence, trafficking of CAR T cells and elimination of extramedullary lesions by CAR T therapy in vivo. (A) Peripheral blood was collected at a serial of time points before and after CAR T infusion. (B) Bone marrow (BM) aspirates and cerebrospinal fluid (CSF) were collected 15 days after CAR T infusion. These samples were analyzed by FACS. (C) Patient 1: Leukemia lesion in the fourth lumbar vertebra before (left panel) and after (right panel) CAR T therapy was demonstrated by PET/CT. FDG uptake in the infiltrating loci disappeared after CAR T therapy. (D) Patient 7: leukemia lesions in the lung were detected before (left panel) and after (right panel) CAR T infusion by PET/CT. Leukemia lesion was regressed after CAR T treatment. Arrows indicate leukemia lesions

As for the persistence, dynamic FCM analysis showed that CAR T cells in PB persisted for ≤20 days and the percentage of CD19 CAR T cells of total lymphocytes decreased rapidly in most of the isolated EM‐ALL patients (Figure 1A, usually more rapidly than those with leukemia blasts in BM or PB or both based on our other studies, in the paper). CAR T cells in patient 6 were barely detectable on day 14 after infusion. CAR T cell percentage of total lymphocytes in the other eight patients also dropped below 2% on day 20 after infusion. This short duration in PB might be due to the lack of CD19 stimulation in PB or BM, further confirming that CD19 positive blasts in BM and PB not only can trigger CD19 CAR T cell proliferation but also help maintain its persistence, which is consistent with the previous view that circulating antigen is an important factor in the proliferation and persistence of CAR T cells in vivo.

The trafficking of CAR T cells to tumor sites is necessary to their antitumor activity [6, 7]. As shown in Figure 1B, approximately 15.8% CD3+CAR19+T cells were also observed in the CSF of patient 2 by FCM on day 15 after infusion, indicating the potential ability of CAR‐T cells trafficking to EM sites, even crossing the blood–brain barrier to eradicate the blasts in the CSF. The high percentage of CD19 CAR T cells in BM aspirates also supported that CD19 CAR T cells could efficiently migrate to the bone marrow (Figure 1B). Consistent with the appearance in the EM sites of CAR T cells, repeated positron emission tomography/computed tomography (PET/CT) showed no evidence of abnormal ^18^F‐fluorodeoxyglucose (FDG) uptake in corresponding lesions of EMDs compared to initial scans (Figure 1C,D). Consequently, all the patients in this study achieved FCM–minimal residual disease (MRD) –negative complete response (CR) on day 30 after CAR‐T cell infusion judged by flow cytometry and PET/CT.

Safety is another concern for CAR T‐cell therapy, especially in patients with CNS involvement. All nine patients had low grades of CRS (Gr1/2), which were self‐limited and needed no specific treatment. Patient 8 developed grade 1 neurotoxicity and successfully recovered with interventions. Other adverse events, especially neurotoxicity in central nervous system leukemia (CNSL) related to CAR T therapy were not observed. These findings suggested that CD19 CAR T cells efficiently eradicated isolated extramedullary leukemia and were well tolerated in these patients, even those patients with CNS involvement.

There is still controversy about whether allo‐HSCT is necessary for r/r B‐ALL patients after CAR T therapy. [8] Some findings have indicated no difference in leukemia‐free survival (LFS) between the patients that underwent allo‐HSCT after CAR T therapy and those who did not, [9] while other studies observed that bridging into allo‐HSCT after CAR T therapy could improve LFS in r/r B‐ALL patients. [10, 11] In this study, as shown in Table S1, once achieved CR after CAR T‐cell treatment, 6/9 of the patients proceeded to consolidative allo‐HSCT. The follow‐up results showed that 3/6 of the allo‐HSCT group remained CR within 17 months after CAR T infusion, and the 1‐year LFS was 66.7%, while in the non‐allo‐HSCT group, both patient 2 and patient 8 relapsed within one year after CAR T infusion and the 1‐year LFS was 33.3%. Despite there being no significant difference due to the small‐sample size, the higher LFS rate in the allo‐HSCT group implies that CAR T therapy bridging to allo‐HSCT can significantly enhance the clinical outcome for isolated EM‐ALL patients.

In summary, based on this study, we envision that CAR T‐cell therapy combining allo‐HSCT is a safe and feasible regimen and offers new hope for these patients. However, only a small number of patients were enrolled in this study, and more cases and further studies are needed to verify these findings.

## CONFLICT OF INTEREST

The authors declare that there is no conflict of interest that could be perceived as prejudicing the impartiality of the research reported.

## AUTHOR CONTRIBUTIONS

Peihua Lu, Bo Chen, and Xiangqun Li designed the clinical trial. Kylan Chen, Xian Zhang, Junfang Yang, Jianwei Zheng, Fei Dong, Yongbo Zhu, and Jiao Yu executed the clinical trial and collected the data. Xiangqun Li analyzed the data and wrote the paper.

## ETHICAL APPROVAL

This study was conducted according to the principles of the Declaration of Helsinki and with the approval of the Ethics Committee of Daopei Hospital.

All the enrolled patients or their families provided informed consent.

## Supporting information

Table S1. Follow‐up after CD19 CAR T therapy
