# Supplementary material for: Successful therapy of chimeric antigen receptor T cells for isolated extramedullary acute lymphoblastic leukemia
Source: EJHaem. 2022 Mar 24;3(2):571–4. doi: 10.1002/jha2.411 (PMC9175730; doi:10.1002/jha2.411)
Supplement: Supplementary file 1 — Table S1. Follow‐up after CD19 CAR T therapy [file JHA2-3-571-s001.docx]

**Supplemental Table 1. Follow-up after CD19 CAR T therapy**

| Patient | Proceed to allo-HSCT (Y /N) | Time from CAR T infusion to allo-HSCT (days) | Type of allo-HSCT | Follow-up time after infusion (Months) | Outcome |
| --- | --- | --- | --- | --- | --- |
| 1 | Y | 57 | Parent 5/10 HLA match | 9 | Gave up due to serious infection |
| 2 | N | N/A | N/A | 5 | Relapsed |
| 3 | N | N/A | N/A | 24 | Relapsed |
| 4 | Y | 66 | Parent 5/10 HLA match | 26 | Remained CR |
| 5 | Y | 56 | Parent 5/10 HLA match | 6 | Died of septic shock |
| 6 | Y | 87 | Parent 3/6 HLA match | 30 | Relapsed |
| 7 | Y | 73 | Parent 8/12 HLA match | 17 | Remained CR |
| 8 | N | N/A | N/A | 4 | Relapsed |
| 9 | Y | 54 | Parent 5/10 HLA match | 19 | Remained CR |

**Abbreviations:** Y, yes; N, no; CAR, chimeric antigen receptor; URD, unrelated donor; allo-HSCT, allogeneic hematopoietic stem cell transplantation; N/A, not applicable; CR，complete response.
